# Promoter architecture of *Drosophila* genes regulated by Myocyte enhancer factor-2

**DOI:** 10.1371/journal.pone.0271554

**Published:** 2022-07-21

**Authors:** Lijing Bu, Richard M. Cripps

**Affiliations:** 1 Department of Biology and Center for Evolutionary and Theoretical Immunology, University of New Mexico, Albuquerque, NM, United States of America; 2 Department of Biology, San Diego State University, San Diego, CA, United States of America; National Institutes of Health, UNITED STATES

## Abstract

To gain understanding into the mechanisms of transcriptional activation of muscle genes, we sought to determine if genes targeted by the myogenic transcription factor Myocyte enhancer factor-2 (MEF2) were enriched for specific core promoter elements. We identified 330 known MEF2 target promoters in *Drosophila*, and analyzed them for for the presence and location of 17 known consensus promoter sequences. As a control, we also searched all *Drosophila* RNA polymerase II-dependent promoters for the same sequences. We found that promoter motifs were readily detected in the MEF2 target dataset, and that many of them were slightly enriched in frequency compared to the control dataset. A prominent sequence over-represented in the MEF2 target genes was NDM2, that appeared in over 50% of MEF2 target genes and was 2.5-fold over-represented in MEF2 targets compared to background. To test the functional significance of NDM2, we identified two promoters containing a single copy of NDM2 plus an upstream MEF2 site, and tested the activity of these promoters in vivo. Both the *sticks and stones* and *Kahuli* fragments showed strong skeletal myoblast-specific expression of a *lacZ* reporter in embryos. However, the timing and level of reporter expression was unaffected when the NDM2 site in either element was mutated. These studies identify variations in promoter architecture for a set of regulated genes compared to all RNA polymerase II-dependent genes, and underline the potential redundancy in the activities of some core promoter elements.

## Introduction

Transcription factors function to influence the accessibility of the core promoter to the transcriptional machinery. Core promoters are characterized by the presence of one or a few of several conserved sequence elements, which in eukaryotes vary in identity depending upon the class of RNA polymerase that transcribes the genes, the location of the sequence relative to the transcription start site, and whether the sequence is restricted to one or both strands of the promoter. For RNA polymerase II-dependent genes, classical studies have underlined the existence and functional significance of promoter elements including the TATA box and downstream promoter element (DPE), each of which, when present, are usually essential for transcription to initiate at another essential consensus sequence, the Initiator (Inr)(reviewed in [1]).

With the advent of whole-genome sequencing and high-throughput transcriptomics, it has been possible to identify the start sites of essentially all transcribed genes in model organisms, and to assess on a global scale the presence and arrangements of promoter sequence motifs. Ohler et al [2] identified ten core promoter elements enriched in *Drosophila* genes, including those described above as well as novel motifs, several of which were subsequently shown to interact with core transcriptional machinery (see for example [3]). Additional core promoter elements were identified by Fitzgerald et al [4] and Down et al [5]. While these studies have identified numerous potentially important core promoter sequences, many of the elements have yet to be functionally evaluated. Interestingly, the range of promoter-enriched sequences include motifs that are restricted to one strand of the DNA such as the TATA box (designated by convention on the coding strand), plus non-directional motifs (NDMs) that can be found on either strand in the promoter.

Given the wide variability of promoter architectures even within RNA polymerase II-dependent genes, an open question had been whether the promoters of genes that are regulated by a certain transcription factor or class of factors are distinct relative to other genes. There is notable evidence for this being the case: of the 15 motifs identified by Fitzgerald et al [4], most showed motif enrichment correlated with a given Gene Ontology function, including the TATA box being over-represented in somatically-expressed genes and under-represented in germline-expressed genes; similarly, Down et al [5] noted that several motifs they identified were associated with specific gene expression patterns in embryos. In addition it has been reported that Hox genes tend to have promoters that are dependent upon the DPE rather than being TATA-dependent [6]. Clearly, high-throughput studies can identify predilections of transcription factors for certain promoter sequences.

To gain further insight into how muscle gene expression is regulated, we sought to determine if targets of the myogenic regulatory factor Myocyte enhancer factor-2 (MEF2) showed characteristic promoter structures. MEF2 was initially characterized as a nuclear factor interacting with an AT-rich sequence in muscle gene promoters and enhancers [7], and subsequently shown to be a member of the MADS domain family of transcription factors that includes serum response factor [8]. While mammalian genomes contain four MEF2-encoding genes that contribute to muscle differentiation (reviewed in [9]), *Drosophila* has a single gene, *Mef2*, that is essential for muscle differentiation [10, 11]. Moreover, *Drosophila* MEF2 was shown to bind to the promoters or enhancers of several myofibrillar protein genes [12, 13]. Genome-wide targets of *Drosophila* MEF2 were identified using chromatin immunoprecipitation-microarray (ChIP-chip) by Sandmann et al [14], which comprehensively underlined the critical role that MEF2 plays in activation of the myogenic program. The authors further validated the targets by demonstrating that a significant number of the identified target genes were directly dependent upon MEF2 for their activation during development.

We used this collection of 169 MEF2 target genes to determine if their promoter architecture showed unique enrichment for core promoter elements. We note that transcription factors are known to bind to both distant enhancers and proximal promoter sequences, and promoters themselves are often minimally active in the absence of enhancer activity. Here we consider the totality of high-confidence MEF2 targets, without consideration of the proximity of the MEF2 sites to the promoter. We found that several canonical promoter sequences were mildly enriched in MEF2 targets compared to a background collection of all RNA polymerase II-dependent genes. More striking was the prominent occurrence in over 40% of MEF2-regulated genes of an element named NDM2. We selected two MEF2 target genes, *sticks and stones* (*sns*) and *Kahuli* (*Kah*) that contain a single iteration of the NDM2 sequence, and determined the functional significance of the element by analyzing the activities of wild-type and NDM2-mutant promoter-*lacZ* reporter constructs in transgenic animals. Both of the control promoter elements showed strong skeletal muscle-specific activity, and activity was not affected when the NDM2 site in either promoter was mutated. Our studies test for the first time the functional importance of this conserved promoter sequence, and its apparent dispensability emphasizes the occurrence of redundant elements within eukaryotic promoters.

## Materials and methods

### Computational methods

We searched for consensus promoter sequences in MEF2 target genes and in a control set of genes. Consensus sequences were the 15 sequences identified by Fitzgerald et al [4], plus the Ohler 10 sequence ([2]; also called the Motif Ten Element, MTE) and a minimal alternative *Drosophila* DPE [15].

Direct MEF2 target genes were derived from Sandmann et al [14]: rather than use all genomic regions shown to bind MEF2 during embryonic stages, we elected to focus upon a high-confidence list of MEF2 targets in order to be more certain of their regulation by MEF2. A total of 169 target genes corresponded to the high confidence assigned gene list in Sheet 1 of S3 Table of Sandmann et al [14], in which ChIP-chip enriched fragments were selected with cutoff of log2 > 0.7 and q < 0.01. These genes in total contained 330 transcription start sites due to several genes having multiple transcription start sites. Background genes used for control analyses were *Drosophila melanogaster* genome sequences (version BDGP6.22), downloaded from the FTP site of Ensemble release 98 (Sept. 2019) [16]. Annotated features coordinators, including gene/transcripts Transcription Start Site (TSS) were obtained using the Ensemble BioMart tool. We enriched for RNA polymerase II-dependent genes by filtering from this data set rRNA, tRNA and other genes according to the Ensemble fly genome annotation obtained through BioMart. There were a total of 1083 promoter sites (115 rRNAs, 312 tRNAs, 335 pseudogenes, 289 snoRNA, 32 snRNAs) excluded, to generate a final list of 21,705 promoters to use for the background control analysis. These promoters included the 330 MEF2-target promoters.

For both MEF2-target and background control promoters, we obtained for analysis sequences corresponding to -200 to +200 relative to the transcription start site, using in-house Perl scripts. Sequences of the MEF2 target promoters are provided in S1 File.

The consensus sequences of the 17 promoter motifs indicated above were scanned across promoter regions of both MEF2 target and background target genes, using Regulatory Sequence Analysis Tools [17]. The number of significant matches (P value < 0.0001 and segment score (weight) > 7.3) for promoter motifs are summarized in Tables 1 and 2. Enrichment for the 17 motifs within [–200, 200] of MEF2 target gene TSS regions were tested using G-test, which were performed on the count of matches for both MEF2 targets and backgrounds using R package “stats” version 3.6.2 [18].

**Table 1:** Enrichment of directional promoter motifs in background and MEF2 target genes.

Motif name	Consensus	Common name	Ohler	Background_+	MEF2_+	Background_+%	MEF2_+%	UniqueGene_Bg	UniqueGene_MEF2	GTest_+	Fold enrichment
DMp1	STATAAA	TATA	3	2,949	38	13.6%	11.5%	4,087	49	0.265	0.847529209
DMp2	TCAGTY	INR	4	5,890	164	27.1%	49.7%	6,996	107	0.000	1.831362865
DMp3	TCATTCG	INR1		559	16	2.6%	4.8%	925	19	0.021	1.882582534
DMp4	KCGGTTSK	DPE	9	813	29	3.7%	8.8%	1,482	40	0.000	2.346136643
DMp5	CGGACGT	DPE1		213	11	1.0%	3.3%	331	15	0.001	3.396713615
DPE	RGWYV			6,966	138	32.1%	41.8%	8,108	118	0.000	1.302991152
DMv1	CARCCCT			701	21	3.2%	6.4%	997	27	0.005	1.970367008
DMv2	TGGYAACR		8	777	17	3.6%	5.2%	1,259	25	0.152	1.439042939
DMv3	CAYCNCTA		7	996	19	4.6%	5.8%	1,310	19	0.332	1.254700621
DMv4	GGYCACAC		1	846	20	3.9%	6.1%	1,138	25	0.062	1.55491081
DMv5	TGGTATTT		6	501	14	2.3%	4.2%	809	16	0.038	1.837960443
Ohler10	CSARCSSAACGS	MTE	10	468	19	2.2%	5.8%	775	29	0.000	2.670260295

Notes: Background_+ and MEF2_+ refer to the frequency of the motif in the Background and MEF2 datasets on the coding (+) strand, respectively. Percent occurrence in all genes in each dataset is also indicated as Background_+% and MEF2_+%. UniqueGene refers to the number of unique genes that contain the motif. GTest_+ shows the p-value for enrichment of the motif in the MEF2 dataset versus the background dataset. Fold enrichment refers to the degree to which the motif is enriched in the MEF2 dataset and is calculated as the ratio of the MEF2_+% over the Background_+%.

**Table 2:** Enrichment of non-directional promoter motifs in background and MEF2 target genes.

Motif	Consensus	Common name	Ohler	Background_All	MEF2_All	Background_All%	MEF2_All%	UniqueGene_Bg	UniqueGene_MEF2	GTest_All	Fold enrichment
NDM1	GAGAGCG	GAGA		1385	64	6.4%	19.4%	1,027	44	0.000	3.039317361
NDM2	CGMYGYCR			4338	167	20.0%	50.6%	2,905	77	0.000	2.532052894
NDM3	GAAAGCT			1403	13	6.5%	3.9%	1,184	11	0.047	0.609440809
NDM4	ATCGATA	DRE	2	3729	45	17.2%	13.6%	2,013	24	0.081	0.793717546
NDM5	CAGCTSWW	E-box	5	3909	88	18.0%	26.7%	2,700	55	0.000	1.480685597

Notes: Background_All and MEF2_All refer to the frequency of the motif in the Background and MEF2 datasets on either strand. Percent occurrence in all genes in each dataset is also indicated as Background_All% and MEF2_All%. UniqueGene refers to the number of unique genes that contain the motif. GTest_All shows the p-value for enrichment of the motif in the MEF2 dataset versus the background dataset. Fold enrichment refers to the degree to which the motif is enriched in the MEF2 dataset and is calculated as the ratio of the MEF2_All% over the Background_All%.

Graphs summarizing the distributions of identified promoter motifs relative to the transcription start sites (bin size = 5 bp) were generated using R package ggplot2 version 3.3.5 [19].

### Recombinant DNA methods

Wild-type and mutant promoter fragments from the *sns* and *Kah* genes were generated using GeneBlocks (IDT). Sequences corresponding to -1000 to +100 relative to the transcription start site were used for *sns* (2R 8797489–8798588), and -640 to +100 were used for *Kah* (3L 589914–589175). Coordinates correspond to Release 6 of the *Drosophila* genome. Promoter elements for both wild-type and mutant sequence were generated as dsDNA, and had engineered restriction sites at either end to facilitate cloning: *Not*I at the 5’ end and *Pst*I at the 3’ end. DNA supplied by the manufacturer was digested with these enzymes (New England Biolabs), and ligated with placZ-attB [20] also cut with the same enzymes. Cutting placZ-attB with *Not*I and *Pst*I removed the minimal heat shock promoter and allowed replacement with the promoter from the gene under investigation. Positive clones were confirmed by sequencing before preparing for injection to generate transgenic animals. The NDM2 site (consensus CGMYGYCR; M = A or C, Y = C or T, R = A or G) was mutated from 5’-cgatgccg to 5’-cCCGgGcg for *sns*, and from 5’-ggacgccg to 5’-ggCcCGGg for *Kah*. Uppercase letters indicates nucleotides changed relative to wild-type. The 5’ nucleotide of the NDM2 sites listed above correspond to nucleotides -77 for *sns* and -78 for *Kah* relative to their respective transcriptional start sites.

### *Drosophila* stocks and crosses

*Drosophila* were maintained at 25°C and raised on Jazz Food (Genesee Scientific). Transgenic lines carrying the promoter-*lacZ* fusion constructs were generated by injecting the constructs described above into the third chromosome Phi C31 Integrase site M{3xP3-RFP.attP}ZH-86Fb [21] present in the Bloomington stock 24749. Injections were carried out by Rainbow Transgenic Flies. Injected larvae (G0 generation) were raised to adulthood and allowed to interbreed. Transgenic offspring were identified in the G1 generation by virtue of orange eye color. Transgenic males and females were interbred to generate homozygous lines, identifiable as those with darker eye color than heterozygotes. Homozygous stocks were expanded for collection of embryos.

### Immunofluorescence

Harvesting and staining of embryos was carried out essentially as described by Patel [22]. The primary antibodies were rabbit anti-MEF2 (1:1000; [23]) and mouse anti-ßGal (1:500; Promega). Secondary antibodies were Alexa conjugated (ThermoFisher) and used at 1:2000. Immunofluorescence was detected using an Olympus FluoView 3000 confocal microscope. Identical microscope settings were maintained when comparing control and mutant reporter lines, and any adjustments to brightness or contrast of the images during preparation of the figures was applied equally to images of control and mutant samples.

## Results

### Presence of directional motifs with precise locations in control and MEF2 target genes

We selected 330 verified MEF2 target promoters for our experimental population of promoters. These promoters were of genes described as high-confidence MEF2 targets by Sandman et al 14, based upon strong enrichment for MEF2 in the ChIP-chip data, and expression in the mesoderm during embryonic development. As a background control we selected 21,705 *Drosophila* promoters. We searched for the presence of each of the 15 motifs identified by Fitzgerald et al (2006), plus the Ohler motif 10 ([2]; also called the MTE), and a more degenerate version of the DPE as described in [15]. For each promoter in each dataset, we determined the location of the candidate motif relative to the transcription start site (TSS), and assessed whether the motif was enriched on the coding or non-coding strands (+ and–strands, respectively).

**Fig 1:**
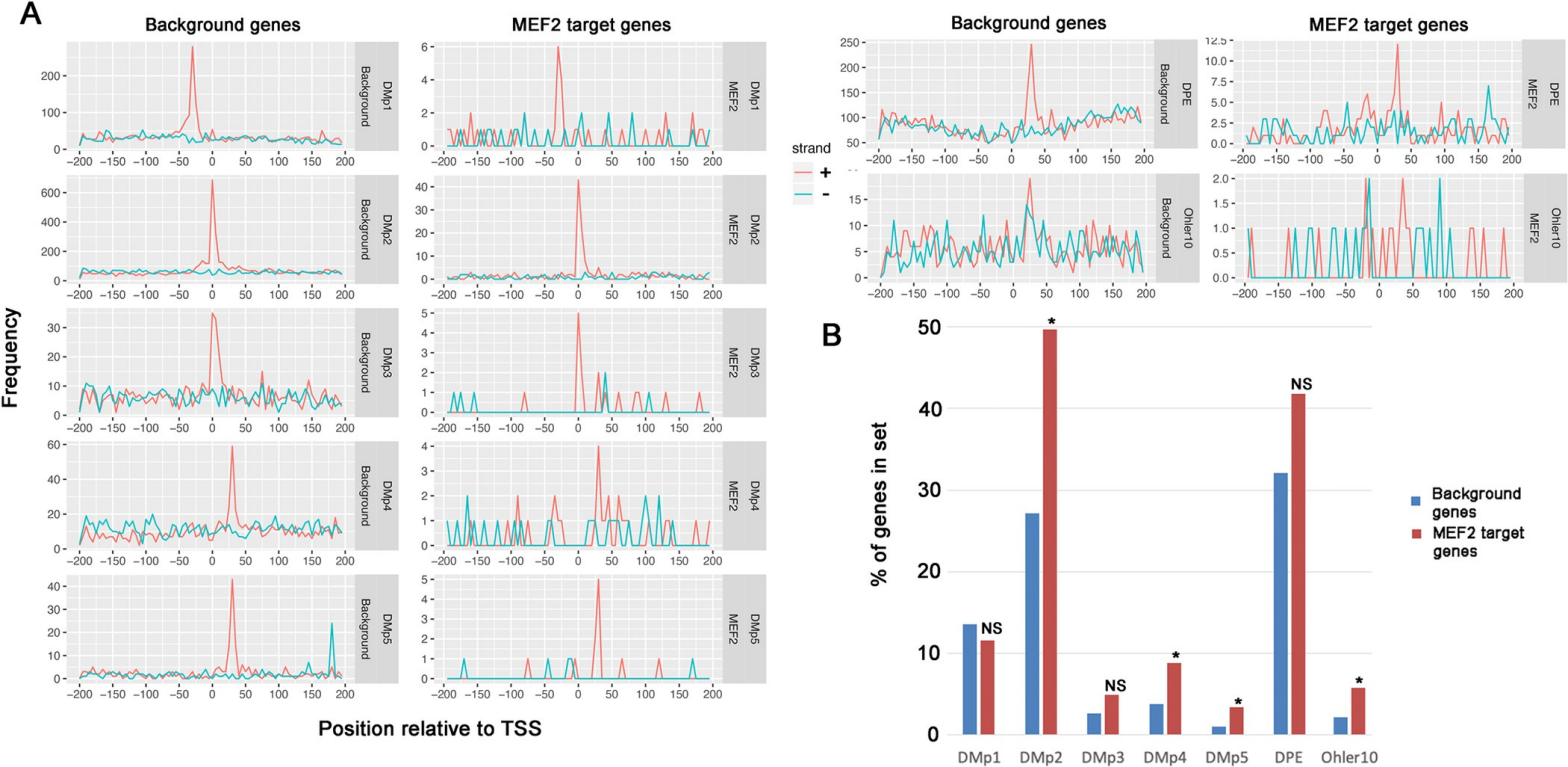
Occurrence of directional promoter motifs with precise locations (DMp) in control and MEF2 target genes. A: Frequency graphs showing the occurrence of each indicated motif in promoters from -200 to +200 relative to the transcription start site. Frequency data, representing the number of times that the element is detected at the indicated location, are shown separately for occurrences on the coding strand (+, red) versus the non-coding strand (-, blue). Graphs representing data from background genes are on the left of each pair of graphs, and data for MEF2 target genes are on the right of each pair. B: Relative frequencies of promoter elements in background and MEF2 datasets. NS, not significantly enriched in the MEF2 dataset; *, significantly P<0.01) enriched in the MEF2 dataset.

For the directional motifs with precise locations (DMp sites) on background genes, we observed enrichment of each motif in the background set of genes at their appropriate locations relative to the transcriptional start site and on the coding strand but not the non-coding strand (Fig 1A, left graphs). For example, the TATA box (DMp1) consensus was enriched on the coding strand (red trace) at -30 relative to the TSS, but was not enriched on the non-coding strand (blue trace). Results for other directional motifs were also generally consistent with prior observations. We noted that the Ohler 10 motif, while being enriched at the appropriate location of ~+25 on the coding strand, showed a noisy signal from -200 to +200, and showed some enrichment at the same location on the non-coding strand. The latter observation likely results from the consensus having some degree of symmetry. Overall, these observations confirmed prior results and importantly demonstrated that our search protocol was effective.

When we searched for the presence of these directional elements with precise locations in the MEF2 target gene dataset, we observed overall similar enrichments on the coding strands compared to the background gene set, and at the same locations relative to the TSS (Fig 1A, right graphs). These data were generally noisier than for the background dataset due to the more restricted number of genes, and as a result enrichment for the Ohler 10 motif was not clearly observed.

We next determined if the occurrence of any of these promoter elements was enriched or diminished in the MEF2 dataset compared to the background. Interestingly, several motifs were found at greater frequencies in the MEF2 targets genes (Fig 1B; also see subsequent figures), and this enrichment was significant for DMp2 (Inr), 4 (DPE) and 5 (DPE2), and for Ohler10/MTE (Table 1). By contrast, we observed that the TATA box (DMp1) was observed at approximately the same frequency in the MEF2 target genes compared to the control dataset. We note that both the DPE and Ohler10 generally require a consensus Inr [3, 24], therefore their coordinated enrichment in the data underlines their functional dependencies.

Overall, our findings demonstrated that in general the architecture of MEF2 target genes does not differ significantly from that of background genes, but that there is differential enrichment of specific motifs.

### Presence of directional motifs with variable locations in control and MEF2 target genes

**Fig 2:**
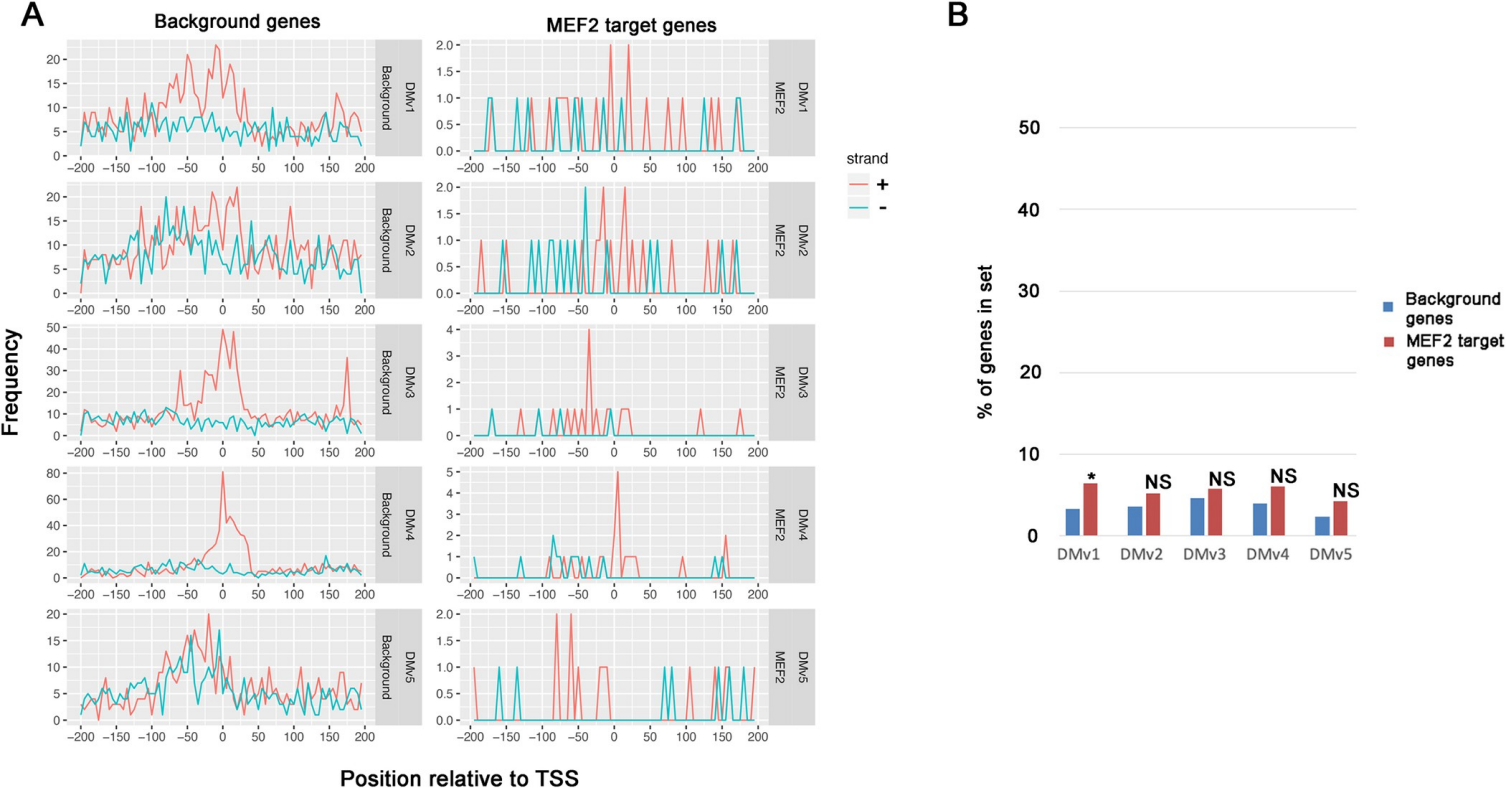
Occurrence of directional promoter motifs with variable locations (DMv) in control and MEF2 target genes. A: Frequency graphs showing the occurrence of each indicated motif in promoters from -200 to +200 relative to the transcription start site. Frequency data, representing the number of times that the element is detected at the indicated location, are shown separately for occurrences on the coding strand (+, red) versus the non-coding strand (-, blue). Graphs representing data from background genes are on the left of each pair of graphs, and MEF2 target genes are on the right of each pair. B: Relative frequencies of promoter elements in background and MEF2 datasets. NS, not significantly enriched in the MEF2 dataset; *, significantly P<0.01) enriched in the MEF2 dataset.

We next performed the same analysis for promoter motifs that have imprecise locations relative to the transcription start site, but that are nevertheless found on the coding strand and not the non-coding strand (DMv motifs). In general, these motifs were observed at lower frequencies in the background dataset (Fig 2A left graphs, compare the *y*-axis numbers with Fig 1A), but were nevertheless observed at their predicted locations. DMv5 also showed some enrichment on the non-coding strand, however in this case the consensus is non-palindromic therefore this may identify DMv5 as a partially non-directional motif. When analyzing the MEF2 target gene dataset, we observed essentially the same results as for controls, although the small sample size of the MEF2 target genes compared to controls meant that the number of genes containing these motifs was small (Fig 2A, right graphs). Given this observation, plus the relative scarcity of the DMv motifs in promoters in general, it was not always possible to definitively conclude that a specific motif was enriched in the MEF2-dependent genes, and enrichment was only significant for Dmv1 (Fig 2B, Table 1).

### Presence of non-directional motifs in control and MEF2 target genes

**Fig 3:**
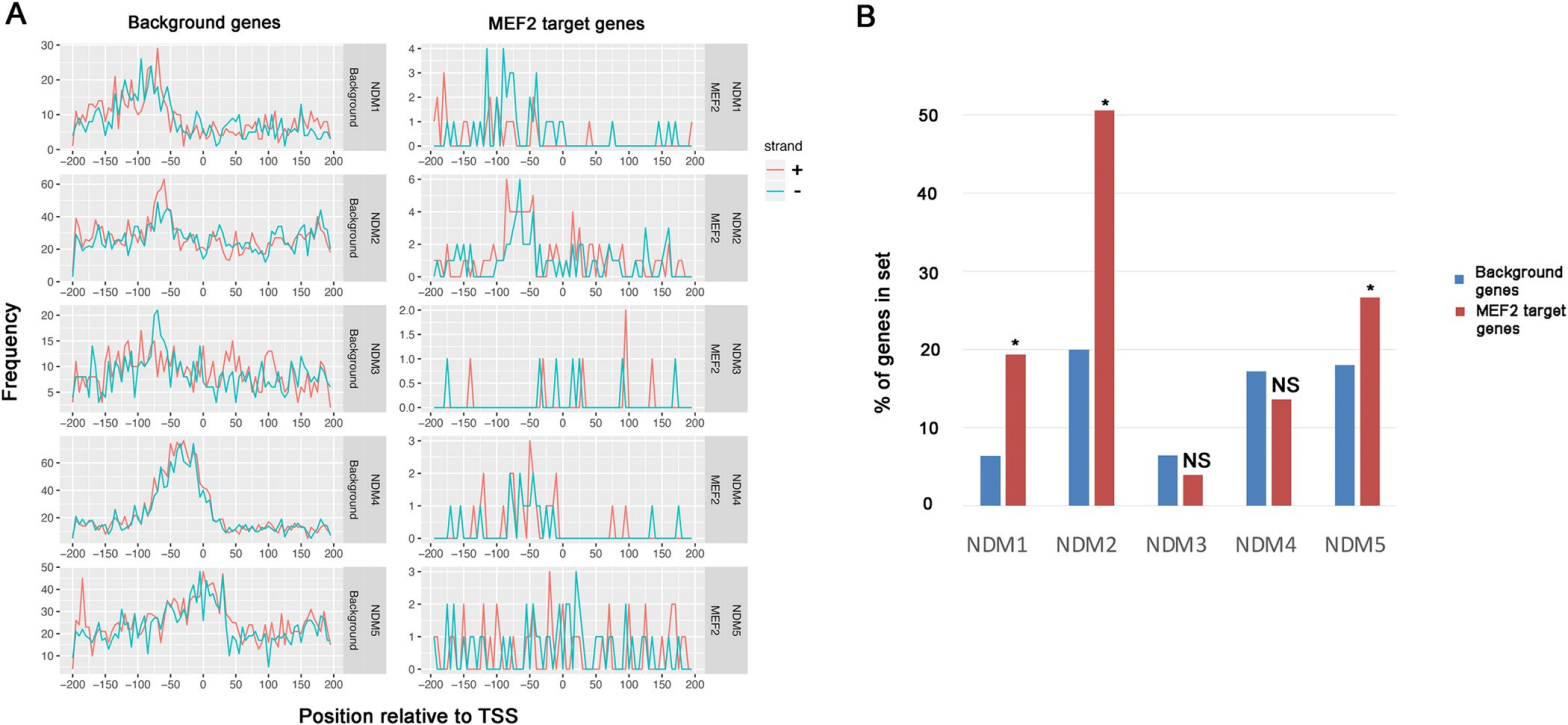
Occurrence of non-directional promoter motifs (NDMs) in control and MEF2 target genes. A: Frequency graphs showing the occurrence of each indicated motif in promoters from -200 to +200 relative to the transcription start site. Frequency data, representing the number of times that the element is detected at the indicated location, are shown separately for occurrences on the coding strand (+, red) versus the non-coding strand (-, blue). Graphs representing data from background genes are on the left of each pair of graphs, and MEF2 target genes are on the right of each pair. B: Relative frequencies of promoter elements in background and MEF2 datasets. NS, not significantly enriched in the MEF2 dataset; *, significantly P<0.01) enriched in the MEF2 dataset.

The third class of promoter motifs characterized by Fitzgerald et al [4] were those observed with equivalent frequency on either strand of the promoter, albeit with relatively restricted locations (NDM motifs). Our confirmatory analysis identified these motifs and their distributions in background promoters (Fig 3A, left graphs). For the MEF2 target genes (Fig 3A, right graphs), NDM3 occurred at sufficiently low frequencies that any enrichment along the promoter was not readily apparent, but all other motifs were observed within the MEF2 target gene promoter set, and enriched at the canonical location relative to the TSS.

Interestingly, when we compared the relative frequencies of the NDMs in MEF2 target promoters compared to background, NDM1 and NDM2 were highly enriched relative to the background promoter set (Fig 3B and Table 2). NDM1 was enriched 3.0-fold in MEF2 target promoters, and NDM2 occurred 2.5 times more frequently in MEF2 target genes (Fig 3B). This result suggested that these motifs might contribute to MEF2 function and thereby muscle gene expression. Overall for our computational analysis, we were able to detect the presence of most canonical promoter motifs enriched at the appropriate locations in the promoters of MEF2 target genes, and several motifs were significantly enriched in frequency in the MEF2 target dataset compared to controls.

### The NDM2 sequence is dispensable for the embryonic activity of two muscle promoters

While NDM1 is thought to interact with GAGA factor, the role of NDM2 in gene expression has not been investigated. NDM2 was observed in the -100 to -50 region of 74 of 169 tested genes (44%), with 38 of these genes containing a single predicted NDM2 site. Given this enrichment for NDM2 sites in MEF2-regulated genes, we hypothesized that activation of these promoters by MEF2 might be dependent upon the NMD2 motif, and tested if NDM2 was required for activity of *Drosophila* muscle promoters. To achieve this, we first identified MEF2 target genes that contained single NDM2 sites in their promoters so that we would be able to remove NDM2 function while making minimal changes to the promoter. In addition, since the basal promoters are unlikely to be active in the absence of tissue-specific enhancers, we also selected genes that had a consensus MEF2 binding site in proximity to the promoter. Through this approach we selected the genes *sticks and stones* (*sns*) and *Kahuli* (*Kah*). The *sns* gene encodes a transmembrane protein expressed in fusion-competent myoblasts that is essential for myoblast fusion [25]. While *sns* expression is still detected in *Mef2* mutant embryos [25], we still consider this a MEF2 target gene based upon the robust ChIP-chip data indicating binding of MEF2 to *sns* at multiple stages of embryonic development [14]. *Kah* encodes a relatively uncharacterized DNA binding protein expressed in skeletal myoblasts [26]. For each gene we inserted the promoter region, plus contiguous upstream sequence containing the MEF2 site, into a promoter-less *lacZ* reporter and generated transgenic lines carrying the constructs.

**Fig 4:**
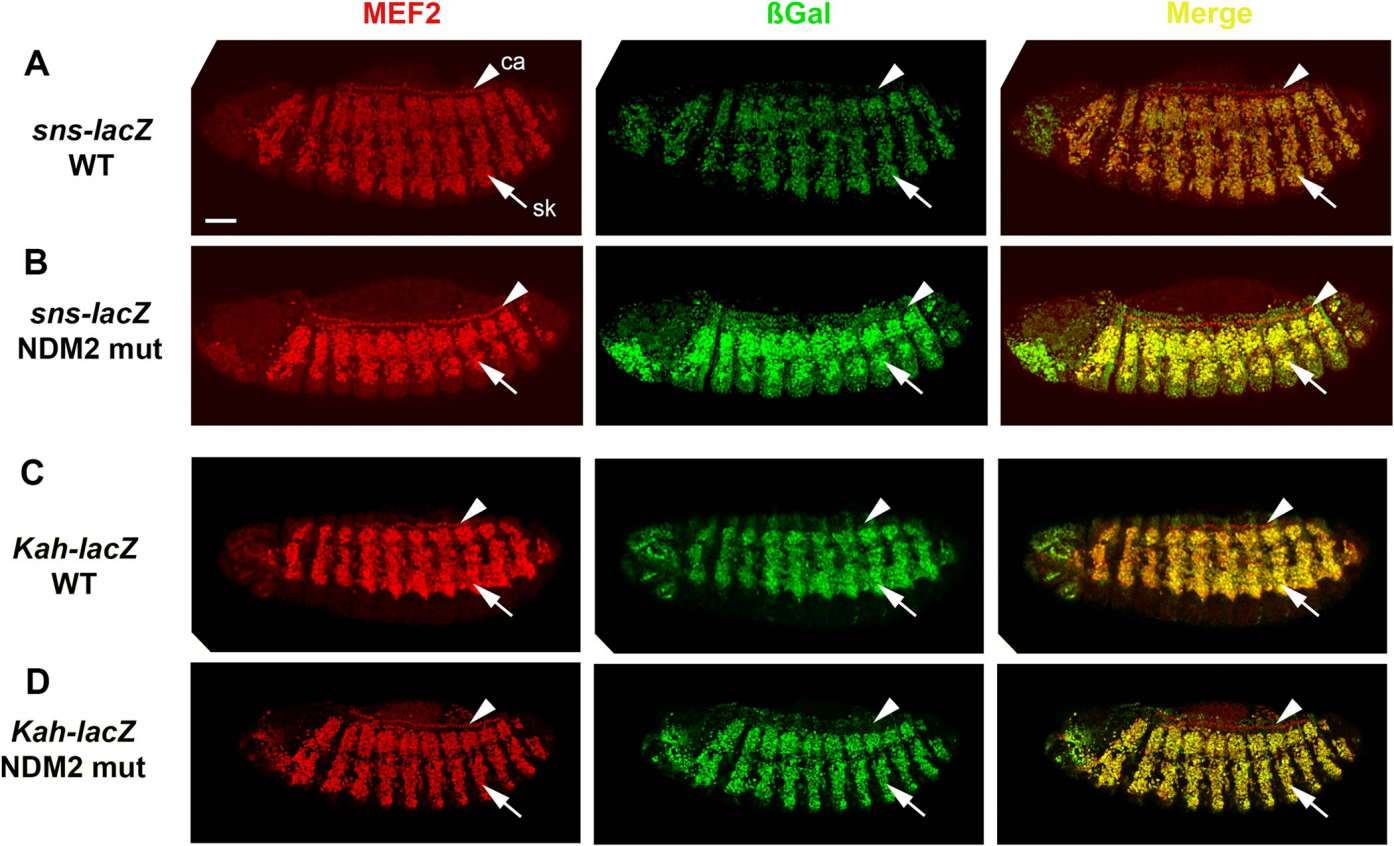
NDM2 is dispensable for the activity of two myoblast-specific promoters. Stage 13 embryos are shown stained for accumulation of MEF2 as a marker of mesodermal cells (red), and ßGal as a reporter for expression of the indicated promoter-*lacZ* transgenes (green). Arrows indicate skeletal muscle myoblasts that express the *lacZ* reporter (sk), and arrowheads indicate cardiac cells (ca) that are ßGal-negative. Bar, 75μm.

For both *sns-lacZ* and *Kah-lacZ* wild-type constructs, we observed robust myoblast-specific reporter expression (Fig 4A and 4C) that was detected in skeletal myoblasts (arrows in Fig 4) but not in cardiac precursors (arrowheads in Fig 4), consistent with the established expression of the parent genes. This confirmed that we had included in the transgenic constructs sufficient enhancer and endogenous promoter sequences for tissue-specific expression.

To determine the functional significance of the NDM2 site in each promoter, we next generated identical constructs except that the NDM2 sites were mutated (see Materials and Methods for details). The mutant transgenic constructs were inserted into the same genomic landing site as the wild-type constructs, therefore there should not be any genomic position effects upon levels nor patterns of gene expression when comparing control and mutant constructs. Despite the mutation of the NDM2 sites in these constructs, both of the mutant reporters showed similar levels and patterns of expression when compared to the wild-type constructs (Fig 4B and 4D), indicating that the NDM2 sites were largely dispensable for expression at this stage of development.

## Discussion

In this manuscript, we determined if canonical core promoter elements were differentially enriched or diminished in target genes of the myogenic regulator MEF2. In general, of all the promoter motifs that have been characterized, we observed their occurrence in at least some MEF2 target genes. We also observed that several promoter motifs were mildly enriched in relative frequency within the MEF2 target gene dataset compared to a control set of *Drosophila* promoters. Finally, we observed that the NDM2 motif was notably abundant and enriched in MEF2 target genes, however we were unable to identify a function for this sequence using in vivo reporters.

The overall enrichment for several motifs in the MEF2 target gene dataset compared to background was notable. Genes whose transcription is mediated by RNA polymerase I or III have highly divergent promoter organizations [27, 28] that do not contain many of the canonical elements that we analyze here. However, those genes are not included in the background dataset, which focuses solely upon genes that are transcribed by RNA polymerase II. Therefore a prevalence of genes regulated by other RNA polymerases is not a contributor to the pattern of enrichment that we observed.

One possible explanation for motif enrichment in MEF2 target genes is that MEF2 target genes are by nature regulated genes, whereas the larger background dataset includes both regulated and housekeeping genes. A recent analysis of genes expressed during embryonic development in *Drosophila* revealed that the DPE, Inr and Ohler 10/MTE are more prominently associated with the promoters of regulated genes expressed later in development [29], which would include MEF2-regulated genes. Indeed, these motifs are certainly enriched in MEF2 target gene promoters (Fig 1 and Table 1). In addition, the same study noted that housekeeping gene promoters and the promoters of genes expressed early during development are generally enriched for DMv3, DMv4, DMv5, NDM4 and NDM5 [29]; interestingly, of these motifs only DMv4 and NDM5 are enriched in the MEF2 target genes. Therefore, an enrichment of housekeeping gene promoters in the background dataset may explain the enrichment for a subset of motifs in the MEF2 dataset.

The enrichment of NDM1 in MEF2-dependent promoters is interesting given that this sequence is commonly known as the GAGA site and binds factors generally associated with transcriptional pausing [30]. One notable facet of MEF2 biology is that it accumulates in myoblasts prior to the observable activation of its target genes [10]. It is feasible that MEF2 target genes are bound by MEF2 in myoblasts, but transcription from the associated promoters is paused until the onset of myogenic differentiation. A role for the GAGA sites and GAGA factor in this model could be readily tested using a similar mutational approach to that used here.

Our mutational analysis did not reveal a critical role for the NDM2 sequence in the expression of *sns* and *Kah* promoter-*lacZ* reporters. While this may point to an insignificant role for this sequence in controlling gene expression, its retention and presence in a large proportion of promoters–from *Drosophila* to humans—indicates that it is indeed functionally important. There are several possible explanations for our failure to uncover its role. One possibility is that the promoters that we chose have additional sequences that could fulfill the function of NDM2. While we selected only genes that had one iteration of the sequence it is possible that additional non-consensus sites are present. A second possibility is that NDM2 functions redundantly with other promoter elements. Either of these two possibilities would provide a mechanism to compensate for the loss of the site that we mutated. Another possibility is that NDM2 functions at a different stage of development to that which is analyzed here. We did not observe any alteration in the stages nor levels of expression of the mutant constructs compared to wild-type, yet it is possible that subtle alterations were not readily detectable.

What is the function of the NDM2 sequence? We retain the hypothesis that NDM2 interacts with a component of the transcriptional machinery. While sequences more proximal to the TSS generally interact with components of the RNA polymerase II holoenzyme, the slightly more remote enrichment for NDM2 around -70 suggests that it may interact with a distinct factor. Indeed, the NDM2 location is bracketed by the locations for NDM4/DRE (around -40) and NDM1/GAGA (-75 to -125), each of which are bound by factors other than the holoenzyme [31, 32]. Future studies will be aimed towards identifying the factor that interacts with NDM2.

## Supporting information

S1 File Sequences of MEF2 target promoters used in this study, from -200 to +200 relative to the transcription start site. (FASTA)
